# Antibacterial Ability of Sodium Hypochlorite Activated with PUI vs. XPF File against Bacteria Growth on *Enterococcus faecalis* Mature Biofilm

**DOI:** 10.3390/dj9060067

**Published:** 2021-06-10

**Authors:** Nerea Herce-Ros, Alejandro Álvarez-Sagües, Laura Álvarez-Losa, Estanislao Nistal-Villan, Ulises Amador, Jesús Presa, Magdalena Azabal

**Affiliations:** 1 Dentistry Department, Faculty of Medicine, San Pablo CEU University, 28668 Madrid, Spain; alejandroalvarez.sag@gmail.com (A.Á.-S.); magdalena.azabalarroyo@ceu.es (M.A.); 2 Microbiology Section, Department of Pharmaceutical and Health Sciences, Faculty of Pharmacy, San Pablo CEU University, 28668 Madrid, Spain; estanislao.nistalvillan@ceu.es; 3 Department of Chemistry and Biochemistry, Faculty of Pharmacy, San Pablo CEU University, 28668 Madrid, Spain; uamador@ceu.es; 4 Independent Researcher, 28003 Madrid, Spain; jesus_l_presa@yahoo.es

**Keywords:** root canal irrigation, sodium hypochlorite (NaOCl), *Enterococcus faecalis* biofilm, passive ultrasonic activation (PUI), XP-endo^®^ Finisher (XPF)

## Abstract

The objectives of the present study were to assess the antibacterial effectiveness of two sodium hypochlorite (NaOCl) concentrations (2.5% and 5.25%) activated by means of two techniques, passive ultrasonic irrigation (PUI) and XP-endo^®^ Finisher (FKG Dentaire SA, La Chaux-de-Fonds, Switzerland) (XPF) against bacteria growth in intracanal mature biofilm. Our aim was to determine if the effect of heating up NaOCl at body temperature (BT) contributed to an improvement of the efficacy of XPF. Sixty-two single-canal human roots previously instrumented were infected with *E. faecalis* inoculum at 0.5 McFarland and incubated at 37 °C for two weeks. Twelve specimens were randomly selected as positive control, and the remaining fifty were divided into five experimental groups (*n* = 10). The first two were irrigated with 2.5 vs. 5.25% NaOCl at room temperature (RT), activated with PUI, and the other three were irrigated with XPF. Of these three, two were irrigated using 2.5 vs. 5.25% NaOCl at RT and one was irrigated with 5.25% NaOCl at BT. Our results showed that NaOCl was effective in biofilm removal for all experimental groups (*p* > 0.05), especially in the groups irrigated with 5.25% NaOCl at room temperature (RT) activated with PUI and the group treated with 5.25% NaOCl at BT with XPF. These groups were the most successful ones (*p* < 0.001). NaOCl, activated with XPF, was as effective as PUI in biofilm removal from the apical third of the canal when it was used at higher concentration and heated up. This study indicates that XPF only reached the efficacy of PUI when NaOCl was heated up.

## 1. Introduction

The prognosis of endodontically treated teeth with apical periodontitis is highly dependent on the removal of biofilm from inaccessible areas of the root canal system [1]. One of the most frequent bacterial species isolated in this type of biofilm is *Enterococcus faecalis*, which is capable of invading the root canal and forming biofilm alone, even in adverse conditions [2]. For this reason, the presence of this type of bacteria has a negative influence on treatment and prognosis, and it is essential to eliminate it during the therapeutic procedure [3,4].

Additionally, one of the factors which may hinder root canal disinfection is the complexity of the root anatomy, such as multiple macroscopic canals and thousands of microscopic dentinal tubules covering the canal walls [5,6,7]. This complexity is especially striking at the apical third, where frequent accessory canals and multiple foramina often create a delta-shaped termination [8,9].

Whereas endodontic files facilitate the cleaning of macroscopic areas, irrigating solutions may help to reduce the number of microorganisms in the whole root canal system, including in dentinal tubules. However, some irrigating agents which have proved effective during in vitro tests are less efficient in vivo as a consequence of the great influence that environmental conditions have on both the mass and structure of biofilm in the root canal [10,11,12].

At present the irrigant most commonly used during endodontic treatment is NaOCl due to its antibacterial and proteolytic properties. However, it is essential to activate it during the procedure in order to impel it through the whole root canal system and to force it to penetrate into the dentinal tubules. The use of ultrasound with passive technique (PUI) after instrumentation is one of the methods which best achieves this aim [13,14].

Nevertheless, newly designed irrigation systems such as XP-endo^®^ Finisher (XPF), offer a competitive alternative to ultrasounds. XPF is an ISO 25/.00 rotary instrument made of nickel–titanium MaxWire^®^ alloy (FKG Dentaire SA Crêt-du-Locle 4. CH-2304 La Chaux-de-Fonds, Switzerland), which at body temperature becomes more malleable. This effect allows the file to contract and expand following canal irregularities. In this way, when this file is used after instrumentation the turbulence created by its free movement not only improves wall cleaning but also increases the penetration of irrigating agents into the dentinal tubules [15,16,17].

For these reasons, both systems were selected to carry out this in vitro study. Our aim was to compare their efficacy on the elimination of mature *E. faecalis* bacterial biofilm on human roots by using NaOCl at two different concentrations, 5.25% (the highest biocompatible one) and 2.5% (the one most commonly used) [18].

In the case of XPF, an additional group was included, which heating the 5.25% NaOCl up to BT to ensure that the file was able to turn into the martensitic phase and allow the irrigant to reach the deepest areas of the canal [19,20].

There are few studies that use NaOCl heated up at BT when using XPF as an irrigant activation technique and, at the same time, compare it with PUI for biofilm removal. Taking into account that one of the main causes of endodontic failure on necrotic teeth is the persistence of biofilm within the most apical canal sections, we consider that this newly designed instrument could be a good alternative to ultrasound, and have tested it with different temperature and NaOCl concentrations.

## 2. Materials and Methods

### 2.1. Specimen Selection and Preparation

About 80 extracted human teeth were collected from an anonymous pool of teeth of San Pablo CEU University in order to ensure the 62 valid teeth after radiological assessment required for our study. For this reason, informed consent is not available. The approval for the study was obtained from the Research Ethics Committee of San Pablo CEU University (certificate 340/19/14), who determined that it complies with the essential ethical requirements demanded in this area (Annex 1).

Crowns were sectioned by using a diamond disc (Brasseler USA^®^ Dental One Brasseler Boulevard Savannah, GA 31419) to standardize the root length (about 15 mm).

Preoperative radiographs were taken in buco–lingual (B–L) and mesio–distal (M–D) projections to assure that the 62 selected teeth had one single canal and were free of any type of pathology or open apex.

Twelve specimens were randomly selected as positive control and the remaining fifty were assigned into the five different experimental groups (*n* = 10), depending on NaOCl concentration, activation technique, and irrigant temperature. The first group was irrigated with 5.25% NaOCl at RT activated with PUI, the second one with 2.5% NaOCl at RT and PUI, the third one with 5.25% NaOCl at RT and XPF, the fourth one with 2.5% NaOCl solution at RT and XPF, and the last one with 5.25% NaOCl at BT and XPF.

The group irrigated with 5.25% NaOCl at BT activated with PUI has not been considered, as according to Van der Sluis [21] when using PUI for 30 s, ultrasound contributes to rising the intracanal temperature from 37 up to 45 °C close to the instrument tip and up to 37 °C away from it, due to the cavitation effect of ultrasound.

Two roots from the positive control were kept to be analyzed by Scanning Electron Microscopy (SEM) in order to confirm the presence of biofilm in the canal walls after the bacteria inoculation and colonization.

Concerning root canal preparation, the working length (WL) was determined at 1 mm from the apical foramen (AF) by using a stainless-steel K-file size #10. Apical patency was performed using a K-flexofile (Dentsply Maillefer) size #15. The instrumentation was followed by the use of Proglider^®^ and WaveOne^®^ Gold Primary files (Dentsply Maillefer), using distilled water as an irrigator. Finally, the roots were sealed with nail varnish in order to emulate the periodontium.

### 2.2. Inoculum Preparation

*Enterococcus faecalis* was obtained from the CECT (Spanish Type Culture Collection). It was cultured on Slanetz–Bartley agar plates with 1% sodium azide, incubated at 38 °C for 24 h and subjected to several successive passes for stabilization. Fresh cultures were prepared two days before the inoculation. The inoculum was obtained by diluting colonies in sterile physiological saline until reaching 0.5 McFarland concentration.

### 2.3. Specimen Inoculation

Each specimen was introduced in a sterilized Eppendorf tube with 500 mL of brain heart infusion culture (BHI) and autoclaved at 120 °C and 1.5 atm for 20 min.

During the tests carried out to determine the final protocol, contamination in neither the BHI culture nor in the dentin extracted from the canals was observed in any the teeth autoclaved as mentioned above after being incubated for two weeks.

Immediately afterwards, 50 μL of *E. faecalis* was pipetted into all root canals, working in a laminar flow cabinet. This procedure not only facilitated the entry of bacteria into the canal, but also aided their penetration into the dentinal tubules. After two weeks, two samples were longitudinally sectioned, making a small groove with a 0.19 mm diamond disc and splitting them into two slices with the aid of a steel blade. Immediately afterwards, both slices were covered with an ionic liquid1-butyl-3-methylimidazolium bis-(trifluorometha-nesulfonyl) (Sigma–Aldrich 711713) and left to dry for 15 min [22]. The excess liquid was removed by means of a filter paper before SEM analysis in order to confirm the bacterial colonization and biofilm formation, as Siqueira et al. described in their experimental work [23].

### 2.4. Specimen Disinfection Treatment

During each complete procedure, all of the specimens were irrigated with the same volume of NaOCl (4.5 mL) and for an equal retention time (1 min), using a sterile syringe and a 30 G side-vented needle (ProRinse^®^, Dentsply Tulsa Dental) placed 2 mm from the AF. At the end of each treatment, the NaOCl was neutralized with a 5% sodium thiosulfate solution, using the same volume as the previous NaOCl replenishment and applying it with the same technique to avoid post-treatment NaOCl activity, following the procedure carried out by Radcliffe et al. in their experimental work [24].

PUI activation was performed with an IrriSafe^®^ ultrasonic tip (IRR 25/25) operated with an ultrasonic device (Saletec, Acteon) set at power 5. The irrigant was activated in three twenty-second cycles (flow rate 0.075 cm^3^/s), placing the tip 1 mm from the AF and using 1.5 mL NaOCl for each replenishment.

The irrigant activation with XPF was performed with a VDW Silver motor, set at 1 Ncm and 800 rpm, with a gentle in-and-out motion following the manufacturer’s recommendations [19] and using 2.25 mL NaOCl replenishment each time (flow rate 0.075 cm^3^/s). The irrigant was activated in two thirty-second cycles, placing the tip 1 mm from the AF. In the case of the group irrigated with 5.25% NaOCl at BT, a test tube containing the irrigant was immersed in a bottle warmer with water heated up to 37 °C. We confirmed the right NaOCl temperature by means of a laser thermometer during the whole procedure.

### 2.5. Dentin Extraction from the Root Canal and Bacteria Quantification

After finishing each disinfection treatment, about 20 mg of dentinal chips were extracted from the apical third of the canal by using a sterile Protaper Next^®^ XA file (Dentsply Maillefer) inserted through the AF up to a depth of 4 mm. This material was weighed to standardize the samples. Next, dentin chips from each specimen were diluted in 1 mL of physiological saline buffer and vortexed for 1 min to facilitate bacteria detachment from dentin.

50 μL from the previous suspensions was pipetted and diluted tenfold, and 100 μL from each dilution was seeded on a Slanetz–Bartley agar plate with 1% sodium azide. These plates were incubated at 37 °C for three days. The colony-forming units (CFU) obtained from each sample allowed to quantify the number of microorganisms remaining in the apical third after each treatment.

### 2.6. Statistical Methods

As the data obtained after CFU counting were overdispersed in relation to the normal model, two non-parametric tests were used: Kruskal–Wallis, to check if there was any significant difference among the groups, and Wilcoxon–Mann–Whitney to analyze the differences between pairs of groups, taking into account that a difference is considered statistically relevant whenever *p* value is lower than 0.05.

## 3. Results

**Figure 1:**
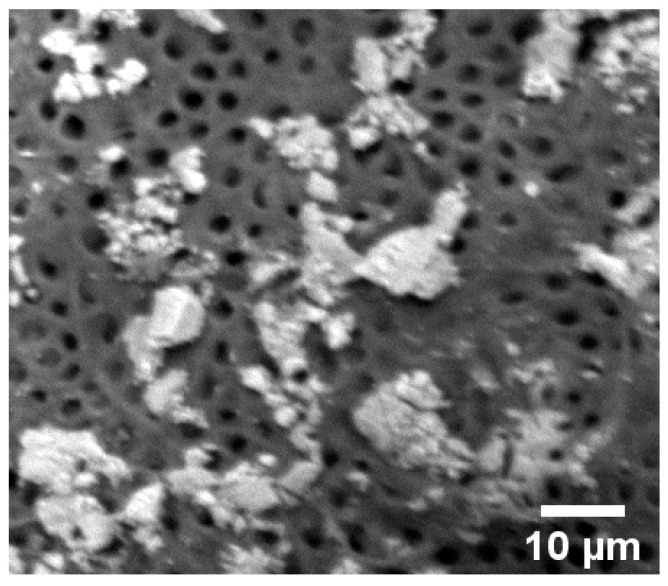
Longitudinal section of root canal, visualized by SEM. The image shows the canal walls with the entrances into the dentinal tubules, individual bacteria, and areas where bacteria have grown forming biofilm. This image was obtained after two weeks of incubation.

In order to visualize the formation of biofilm, dental roots were inoculated with bacteria as indicated in the materials and methods section. After two weeks of incubation, the roots were longitudinally sectioned and the root canals were analyzed by scanning electron microscopy (SEM) (Figure 1).

**Figure 2:**
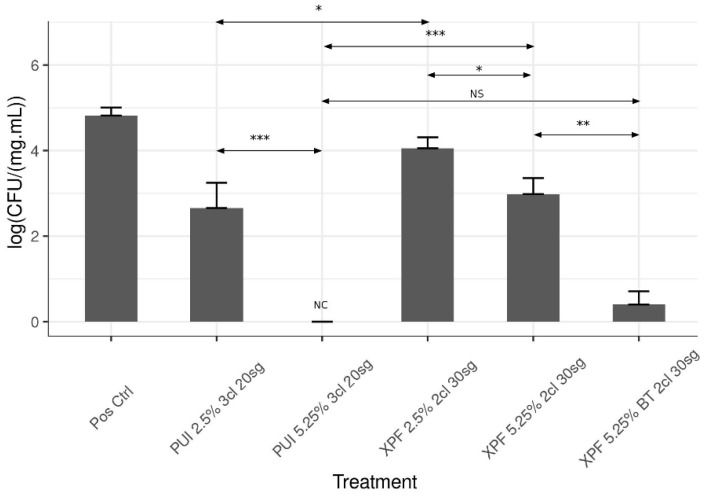
An overview of bacterial count located within the apical third and quantification of bacteria present in the apical third. CFU were standardized with the weight of dentin. The experiments were carried out using at least 10 specimens for each group. The error bar represents the standard error of the mean. The Wilcoxon–Mann–Whitney test was used to evaluate the differences between the pairs of experimental groups: *p* < 0.05 (*), *p* < 0.01 (**), and *p* < 0.001 (***). NS, no significant differences; and NC, no colonies counting.

CFU analysis showed that the treatment in all experimental groups was effective in reducing the mean bacteria count from the apical third of the canal (*p* < 0.05) (Figure 2). At the same concentration of NaOCl, PUI was more effective than XPF when it was used at RT, with *p* = 3.7 × 10^−2^ in the NaOCl 2.5% groups and *p* = 2.3 × 10^−4^ in the NaOCl 5.25% ones. However, the group irrigated with 5.25% NaOCl at BT activated with XPF showed a huge reduction in comparison with the same treatment at RT, reaching a similar efficiency as the one achieved in the group irrigated with 5.25% NaOCl at RT activated with PUI (*p* = 0.168) (Table 1).

## 4. Discussion

This study has analyzed the efficacy of NaOCl at two different concentrations (5.25% vs. 2.5%) in the removal of mature *E. faecalis* biofilm from the apical third using two different activation systems (XPF and PUI) with the aim of determining the most effective procedure.

PUI was used in three twenty-second cycles, placing the tip 1 mm from the AF. A higher number of cycles would not have contributed to a significant reduction of the organic matter or debris, as shown by Duque, Van der Sluis, and Macedo [25,26,27] in their studies.

However, although XPF was also inserted 1 mm from the AF, instead of using it for one minute at a time it was used in two thirty-second sequences. This was done with the aim of achieving a balance between a higher efficiency due to the NaOCl replenishment and a lower malleability of the file as the consequence of a lower frictional heating (Leonardy et al.) [28].

Other researchers, such as Bao et al. [1] and Sasanakul et al. [29] have also stated the higher effectiveness of XPF when using it in three twenty-second sequences instead of for one minute at a time. We have considered that an intermediate approach could have both effects, i.e., file heating and NaOCl replenishment.

The final findings revealed that the samples from the group irrigated with 5.25% NaOCl at RT activated with PUI and the ones irrigated with 5.25% NaOCl at BT activated with XPF showed a higher reduction in the bacteria count, as no CFU were detected in the samples from the first one and in the majority of the samples from the second one, decreasing the efficiency when XPF was used at RT.

The 300-fold CFU reduction observed between the group of samples irrigated with 5.25% NaOCl at BT activated with XPF and the one treated with 5.25% NaOCl at RT activated with XPF could not have been explained only by the NaOCl temperature effect, estimated by Sirtes G et al. [30] in 100-fold per 25 °C, but also by the improvement of the file malleability when turning into its martensitic phase (M-phase), promoting a better distribution of the irrigant throughout the whole root canal system.

This fact was also confirmed in the study published by Pacheco-Yanes et al. [31] with the aim of comparing the irrigant distribution throughout mesial canals joined by an isthmus in lower molars using conventional positive pressure and two activation techniques (PUI and XPF, assessed by means of micro-CT). In this study, all of the procedures were carried out within a laminar flow cabinet under the controlled temperature of 37 °C. Additionally, the NaOCl used as irrigant was heated up to 37 °C by means of a heater (800-Heater, Plas-Labs, Lansing, MI, USA) before use. The authors concluded that XPF promoted better distribution of the irrigant throughout the root canal system, especially in the apical third, when compared to PUI and conventional positive pressure.

With regard to the efficiency of both techniques on biofilm removal, our findings contradict those of Bao et al. [1], who in their study concluded that XPF was more efficient than PUI. This difference might be attributed to the way the experimental design was developed and the technique used for the remaining bacteria assessment by these authors. As in Bao et al., the specimens were longitudinally split into two slices to grow biofilm, and then reassembled and fixed with a wax ball over the root tip before treatment, which could have altered the natural biofilm environment. Additionally, the authors used a qPCR approach technique for bacteria quantification, which does not discriminate between live and dead bacteria and which may have altered the interpretation of the treatment effect on bacteria viability.

Concerning NaOCl concentration, both systems (PUI and XPF) showed higher efficiency with 5.25% NaOCl, concurring with the results of other studies [32,33] which also obtained better antimicrobial efficiency at higher NaOCl concentration. However, other researchers have found no significant differences [34,35], most likely because the lower concentration was compensated by a higher volume of irrigant. This fact is also discussed in the review by Căpută et al. [36], who states that other factors related to the irrigant, such as volume or retention time, must be considered during the canal disinfection.

Our research has shown a partial bacteria reduction in the groups irrigated at the lower concentration (2.5%), being more effective in the group activated with PUI. However, this slightly significant difference between PUI and XPF could not have been relevant in a clinical endodontic treatment when NaOCl is used at RT as, according to the in vivo study carried out by De Hemptinne et al. [37]. In this case, the temperature that the irrigant would reach within the canal would be at an intermediate point between RT and BT (32–33 °C), which could contribute to a better performance of XPF.

For this reason, further in vivo studies would help to clarify if this lower NaOCl concentration would be enough to guarantee the success of the endodontic treatment on infected teeth.

## 5. Conclusions

Within the limitations of this study, it was concluded that the activation of 5.25% NaOCl at RT with PUI was more effective in bacteria reduction and biofilm destruction than the one achieved with XPF in the same conditions. However, when 5.25% NaOCl was heated up at BT and was activated with XPF, both systems showed similar efficacy. On the other hand, the activation of 2.5% NaOCl was partially efficient when using either PUI or XPF.

In order to try to overcome the limitations of this study, it would be interesting to perform further long-term in vivo studies in infected teeth with chronic apical periodontitis, comparing the effectiveness of XPF to activate NaOCl at BT vs. RT, and assessing the efficacy of the treatment by means of micro-CT with follow-up evaluation.

## Figures and Tables

**Table 1:** Average of log10 CFU/mLxmg): SD, standard deviation; SE, standard error. Bacteria percentage reduction and the positive control *p* value comparation of *E. faecalis* in the AF after chemo–mechanical treatment.

Group	n	After Treatment (CFU/mLxmg)	SD	SE	Percentage Reduction	*p* Value
Positive Control	10	4.82	0.67	0.18		
PUI 2.5% NaOCl (RT)	10	2.65	1.76	0.58	99.31	2.12 × 10^−3^
PUI 5.25% NaOCl (RT)	10	0	0	0	100	3.00 × 10^−6^
XPF 2.5% NaOCl (RT)	10	4.05	0.81	0.25	82.80	3.58 × 10^−2^
XPF 5.25% NaOCl (RT)	10	2.98	0.18	0.37	98.54	1.17 × 10^−3^
XPF 5.25% NaOCl (BT)	10	0.40	0.97	0.30	99.99	4.39 × 10^−5^

## Data Availability

The data set generated and analyzed during the study are available upon reasonable request to the author.
